
JOURNAL INFORMATION
==============================
NLM Title Abbreviation: Atlanta Med Surg J (1884)
NLM Journal ID: 101251623

Atlanta Medical and Surgical Journal (1884)

Publisher: Biomedical Journal Digitization Project

ARTICLE INFORMATION
==============================
PMCID: PMC9199287
PMID: 35829586
Article version: 1
Subjects: Original Communications

Some of the Complications That Are Met with in Obstetrical Practice* Read before the Atlanta Society of Medicine.

Crowe Walter A. M.D. Atlanta, Ga.

Print publication date: 1898 Dec
Volume: 15
Volume Series: New Ser
Issue: 10
First page: 673
Last page: 675
License: This work is free of known copyright restrictions. For more information, please see PMC Back Issue Digitization.
License URL: https://creativecommons.org/publicdomain/mark/1.0/

==============================

entirely. Patient lived out of the city, and had to come in twice weekly to my office. During these visits she would invariably buy morphia. Her husband stated the case to me and decided that, as she was practically cured, he thought it best to stop her visits to the city; in this I agreed with him.

Fourteen months after this patient came to my office and reported that she had had only three or four slight attacks since the last time she had seen me. She had improved very much in general condition and had gained twenty-three pounds in weight.

I will say, in conclusion, that although I do not believe all cases of asthma are due to intranasal pressure, I believe a great many are, and that in a disease as intractable and as difficult to handle as this, the nose should invariably be investigated and examined with great care.

---

### SOME OF THE COMPLICATIONS THAT ARE MET WITH IN OBSTETRICAL PRACTICE.*

By WALTER A. CROWE, M.D.,

ATLANTA, GA.

In the short paper that I have presumed to read to-night, I will give the results of my own observations and opinions, based on personal experience in this class of work; and for this reason I hope to elicit a free discussion and opinion from the fellows of the Society. I propose to discuss the mechanical management of labor as met with under certain conditions.

For the obstetrician to render a safe and intelligent service in these cases, it is absolutely necessary that he should have a clear conception of the most minute detail in the mechanism of labor, and to be of more service than that of an ordinary "granny," it is imperative that he should make a correct diagnosis as early as possible in regard to both presentation and position of the child. This I confess is not always an easy matter; under certain conditions it is sometimes a most difficult task, but with patience, prac-

*Read before the Atlanta Society of Medicine.

tice and perseverance a fairly accurate opinion can be given. The methods used in diagnosis are abdominal palpation, vaginal examination (digital) and locating the fetal heart sounds.

Abdominal palpation I consider among the most important of these methods, and I am inclined to think the general practitioner has not fully appreciated the amount of diagnostic information to be gained by this method of examination. It is quite difficult for the beginner, which is also true in auscultation of the chest, but by perseverance and practice we are soon able to feel and hear that which we would otherwise fail to recognize.

The most favorable time for abdominal palpation is before labor begins or during the interim of the pains in the early stages. The examination per vaginam is not always satisfactory, and it is with extreme difficulty in some cases that we can learn more than the presentation without giving an anesthetic and introducing a portion or the whole hand into the vagina.

In about 20 per cent. of the vertex presentations the occiput is either right or left posterior, and in from 95 to 98 per cent. of these occipito-posterior cases the occiput will rotate to the front and then labor progresses normally. The conditions that favor this rotation to the front are flexion, good pains, and a good firm pelvic floor. It is the counteracting force of those muscles that make up the pelvic floor—viz.: Levator ani, obturator internus, pyriformis, and coccygeus—that causes the occiput to strike the anterior plane and rotate to the front; otherwise the occiput sinks down into the hollow of the sacrum, and then we have what is called persistent occipito-posterior position, which then becomes a very serious condition to both mother and child. The death-rate to the child runs from 10 to 15 per cent., and it is almost impossible to deliver these cases without more or less damage to the soft parts of the mother.

These cases are necessarily slow and tedious, and are very trying to the strength of the patient if left alone.

The means of relief that we have at our disposal is either high forceps delivery, or to turn and deliver by the feet. Each of these measures has its proper place and is justifiable, but I am loath to recommend the indiscriminate use of the forceps, especially in unskilled hands, where the possibility of doing such serious damage is so great.

I am inclined to consider version as the safer of the two methods in a great many of these cases. I have had two such cases in my practice in the last few months, and delivered without any trouble whatever; in both of these cases the membranes had ruptured and waters escaped. This is not always possible or even advisable on account of the possible danger of rupture of the uterus, but in cases with thin abdominal walls and feeble uterine contractions it is very easy to perform, and with less risk than the high forceps delivery in this same class of cases.

*Rupture of the perineum.*—This is the most common laceration of the birth-canal and leads to a multitude of after ailments that require the services of the gynecologist to promise any hopes of relief or cure. All tears that involve more than the fourchette should be repaired at once, and while satisfactory results can be obtained under favorable conditions, still I am confident that a large number of these cases fail to get the satisfactory union of muscular structure that is so essential to the support and healthy condition of the pelvic organs.

So it demands our most careful attention both in prevention and cure. As a preventive a number of measures have been suggested, such as support of the perineum, application of hot cloths, etc., all of which are of little value in themselves. I believe more can be accomplished by placing the woman on her side when the head begins to press on the outward parts, so as to keep the parts in view, then by proper pressure keeping the shortest diameter of the presenting part within the outlet; also, by retarding too hasty delivery, a great deal can be accomplished, but from my observation I am inclined to the opinion that more tears are caused from the hasty and improper delivery of the shoulders than from the passage of the head. To be able to anticipate and correct these conditions marks out the true worth and ability of the obstetrician, also gives to the patient the intelligent service that is justly expected of any one who assumes the responsibility of a labor case.
